# Serum Immunoglobulin M Concentration Is Positively Related to Metabolic Syndrome in an Adult Population: Tianjin Chronic Low-Grade Systemic Inflammation and Health (TCLSIH) Cohort Study

**DOI:** 10.1371/journal.pone.0088701

**Published:** 2014-02-12

**Authors:** Kun Song, Huanmin Du, Qing Zhang, Chongjin Wang, Yinting Guo, Hongmei Wu, Li Liu, Qiyu Jia, Xing Wang, Hongbin Shi, Shaomei Sun, Kaijun Niu

**Affiliations:** 1 Health Management Center, Tianjin Medical University General Hospital, Tianjin, China; 2 Nutritional Epidemiology Institute, Tianjin Medical University, Tianjin, China; 3 School of Public Health, Tianjin Medical University, Tianjin, China; Sanjay Gandhi Medical Institute, India

## Abstract

Persistent low-grade systemic inflammation has been increasingly recognized as a common pathological process, and an important contributing factor to cardiovascular diseases and its risk factor, metabolic syndrome. Immunoglobulin M is reactive to multiple autoantigens and is inferred to be important for autoimmunity, implying that immunoglobulin M may be a potential risk factor for metabolic syndrome. However, few epidemiological studies are available which are related to this potential link. Therefore, we designed a cross-sectional study of 9,379 subjects to evaluate the relationship between immunoglobulin M and metabolic syndrome in an adult population. Subjects who received health examinations were recruited from the Tianjin Medical University General Hospital-Health Management Center in Tianjin, China. Immunoglobulin M was determined with an immunonephelometric technique. Metabolic syndrome was defined according to the criteria of the American Heart Association scientific statements of 2009. Multiple logistic regression analysis was used to examine the relationships between the quartiles of immunoglobulin M and the prevalence of metabolic syndrome. After adjustment for covariates, the odds ratio of having metabolic syndrome in the fourth quartile compared with the first quartile of immunoglobulin M was 1.19 times for males (95% confidence interval, 1.002–1.41) and 1.39 times for females (95% confidence interval, 1.07–1.80). Immunoglobulin M levels also showed positive relationships with the ratio of elevated triglycerides and reduced high-density lipoprotein cholesterol in males. The study is the first to show that immunoglobulin M is independently related to metabolic syndrome and its individual components (elevated triglycerides and reduced high-density lipoprotein cholesterol) in males, whereas immunoglobulin M is independently related to metabolic syndrome in females but not to its individual components. Further studies are needed to explore the causality and the exact role of immunoglobulin M in metabolic syndrome.

## Introduction

Chronic diseases, such as cardiovascular diseases (CVD), cancer, and age-related diseases have long been considered among the most important global public health issues [1]. CVD are a group of disorders that affect the heart and blood vessels, and remain a major cause of mortality and morbidity worldwide [1]. Metabolic syndrome (MS) is a well-recognized risk factors for CVD, comprised of a constellation of physiological and biochemical abnormalities characterized by disturbances of glucose metabolism, hypertension, dyslipidaemia, and central obesity [2]. Clarifying the common pathological process of MS or CVD is a crucial step toward providing their early prevention and treatment. Persistent chronic low-grade systemic inflammation has been increasingly recognized as a common pathological process and an important contributing factor to MS or CVD [3]–[6].

Over the past few decades, there has been a steep increase in obesity throughout the world [7], [8]. Obesity induces the development of MS [9]. With obesity, many immune cells infiltrate or populate in adipose tissue and promote chronic low-grade inflammation [10]. Furthermore, fat cells, particularly those in the visceral fat, are now considered an immune organ. These cells secrete numerous immune modulating molecules which directly contribute to the development of low-grade inflammation [11], [12]. Obesity also influences specific immune responses mediated by the mechanisms of humoral immunity [13], [14]. From the above, obesity is, due to innate immunity and/or humoral immune responses that trigger autoantibody production, the most important risk factor for inducing a systemic inflammatory response.

On the other hand, Immunoglobulin M (IgM) is the first antibody to be produced during an immune response after an initial antigen encounter, and is the predominant isotype secreted in T-cell independent immune responses [15]. IgM has a low affinity for modified self-components [16]. An increased IgM concentration is reactive to a wide variety of autoantigens, and its levels are found markedly elevated in a series of autoimmune diseases [17]. It is therefore believed to be an important component in autoimmunity [17], [18]. Because obesity induces the development of autoimmunity [13], [14], and is a core factor of MS [19], [20], it is hypothesized that IgM may be a crucial molecular link between the obesity-inducted systemic inflammatory response and MS. However, few epidemiological studies have evaluated the relationships between IgM and MS among the general population [21]. Therefore, it is still unclear whether a higher level of serum IgM concentration is related to a higher prevalence of MS.

This cross-sectional study aimed to investigate how serum IgM concentration is related to the prevalence of MS in an adult population.

## Materials and Methods

### Participants

The Tianjin Chronic Low-grade Systemic Inflammation and Health (TCLSIH) Cohort Study is a large prospective dynamic cohort study focusing on the relationships between chronic low-grade systemic inflammation and the health status of a population living in Tianjin, China. Tianjin is a city of approximately 10.43 million inhabitants, located in the northeastern part of the North China Plain, facing the Bohai Sea [22]. Participants were recruited, while having their annual health examinations at the Tianjin Medical University General Hospital-Health Management Center, the largest and most comprehensive physical examination center in Tianjin.

This cross-sectional study used baseline data from the TCLSIH. During the research period there were 10,015 participants who had received health examinations including serum-immunological tests. We excluded participants who did not complete data collection on any components of MS (n = 41), body height and/or body weight measurements (n = 1), or those with a history of CVD (n = 513) or cancer (n = 81). Owing to these exclusions, the final cross-sectional study population comprised 9,379 participants (mean [standard deviation, SD] age: 46.6 [10.9] years, age range: 25–86 years; males, 60.5%). The blood sample was routinely drawn 12 ml of whole blood for 2 ml of plasma and 10 ml of serum from each subject. The protocol of this study was approved by the Institutional Review Board of the Tianjin Medical University and participants gave written informed consent prior to participation in the study.

### Serum-immunological tests

Serum-immunological tests were measured as a health examination item. Serum levels of immunoglobulins (IgM, IgA, and IgG) were determined by the immunonephelometric technique using the automated IMMAGE 800 immunochemistry system (Beckman Coulter, Brea, CA, USA), and expressed as mg/dL. The detection limit of the assay was: IgM 4.2 mg/dL, IgA 6.7 mg/dL, IgG 33.3 mg/dL; the measurement range was: IgM, 4.2–14,400 mg/dL, IgA 6.7-25,200 mg/dL, IgG 33.3-21,600 mg/dL; and the intra- and inter-assay coefficients of variation (CV) were less than 6% for three classes of immunoglobulins. The manufacturer indicates the following reference intervals for healthy adults: IgM 46–304 mg/dL, IgA 82–453 mg/dL, and IgG 751–1,560 mg/dL.

### Assessment of MS and other variables

Waist circumference was measured at the umbilical level with participants standing and breathing normally. Blood pressure (BP) was measured twice from the upper left arm using a TM-2655P automatic device (A&D CO., Tokyo, Japan) after 5 minutes of rest in a seated position. The mean of these 2 measurements was taken as the BP value. Blood samples for the analysis of fasting blood sugar (FBS) and lipids were collected in siliconized vacuum plastic tubes. FBS was measured by the glucose oxidase method, triglycerides (TG) were measured by enzymatic methods, low-density lipoprotein cholesterol (LDL) was measured by the polyvinyl sulfuric acid precipitation method, and high-density lipoprotein cholesterol (HDL) was measured by the chemical precipitation method using reagents from Roche Diagnostics on an automatic biochemistry analyzer (Roche Cobas 8000 modular analyzer, Mannheim, Germany).

MS was defined in accordance with the criteria of the American Heart Association scientific statements of 2009 [23]. Participants were considered to have MS when they presented three or more of the following components: 1) elevated waist circumference for Chinese individuals (≥85 cm in males; ≥80 cm in females), 2) elevated TG (≥1.7 mmol/L), or drug treatment for elevated TG, 3) reduced HDL (<1.0 mmol/L in males; <1.3 mmol/L in females) or drug treatment for reduced HDL, 4) elevated blood pressure (SBP ≥130 mm Hg and/or DBP ≥85 mm Hg) or antihypertensive drug treatment, 5) elevated fasting glucose (≥5.56 mmol/L) or drug treatment for elevated glucose.

### Assessment of other variables

Anthropometric parameters (height and body weight) were recorded using a standard protocol. Body mass index (BMI) was calculated as weight/height^2^ (kg/m^2^). Sociodemographic variables, including gender and, age were also assessed. A detailed personal and family history of physical illness and current medications were noted from “yes” or “no” responses to relevant questions. Information on alcohol and tobacco use were obtained from a questionnaire survey.

### Statistical analysis

All statistical analyses were performed using the Statistical Analysis System 9.3 edition for Windows (SAS Institute Inc., Cary, NC, USA). Because the prevalence of MS was significantly higher in males, and their IgM serum concentration was also significantly different (see the results section), males and females were analyzed separately in this study. Descriptive data are presented as the mean (range) for continuous variables, and as percentages for categorical variables. Differences in serum IgM levels and the MS prevalence between genders were examined by t-test or chi-squared test, respectively. Because the distribution of serum IgM levels was non-normal, the natural logarithm was applied to normalize the data before analysis of the t-test, and the descriptive data is presented as the geometric mean (95% confidence interval, CI) for it. For further analysis, the prevalence of MS was used as a dependent variable, and the quartiles of IgM as independent variables. For participant characteristics analysis, the differences among IgM categories were examined using analysis of variance (ANOVA) for continuous variables, and logistic regression analysis for proportional variables. Bonferroni-corrected *P* values were used for comparisons between IgM quartiles. Multiple logistic regression analysis was used to examine relationships between IgM categories and the prevalence of MS after adjustment for covariates: age, sex, BMI, smoking status, drinking status, family history of CVD, hypertension, hyperlipidemia, or diabetes, and serum IgA and IgG concentrations. Odds ratio (OR) and a 95% CI were calculated. Moreover, a developed multivariable logistic model was obtained using stepwise variable selection methods applied to the variables with a *P* value of <0.20 based on the univariable analysis. A linear trend across increasing quartiles was tested by using the median value of each quartile as an ordinal variable. All tests were two-tailed and *P*<0.05 was defined as statistically significant.

## Results

In this study, 60.5% of participants were males and 39.5% females, with mean ages (SD) of 46.3 (10.4) and 47.1 (11.6) years, respectively. The overall prevalence of MS was 34.9% (3,277 of 9,379). The prevalence of MS was significantly higher in males than in females (43.0% compared with 22.6%, *P*<0.0001). In contrast, the levels of IgM were significantly lower in males (geometric mean, [95% CI]: 80.4 [79.4–81.4] compared with 111.0 [109.3–112.7] mg/dL, *P*<0.0001).

Characteristics of male participants across quartiles of IgM are presented in **Table 1**. Compared with participants in the lowest quartile of IgM, participants in the upper three quartiles tended to be younger, have lower BMI, waist circumferences, SBP, and DBP, and higher TG, IgA, and IgG. A lower proportion had a family history of CVD or diabetes (*P* for all trends ≤0.05). Otherwise, no significant difference was observed between different quartiles of IgM.

**Table 1:** Male participant characteristics by quartiles of immunoglobulin M (n = 5,673)^a^.

	Quartiles of immunoglobulin M (range, mg/dL)				
	Level 1 (7.2–59.2)	Level 2 (59.3–80.6)	Level 3 (80.7–108.0)	Level 4 (109.0–2080.0)	*P* for trend^b^
	(n = 1,422)	(n = 1,415)	(n = 1,406)	(n = 1,430)	
Age (y)	48.5 (27.0, 81.0)^c^	45.7 (25.0, 85.0)^d^	45.6 (26.0, 86.0)^d^	45.4 (25.0, 85.0)^d^	<0.0001
BMI (kg/m2)	26.3 (17.3, 45.2)	26.3 (16.5, 51.1)	26.0 (16.6, 44.6)^d^	25.8 (15.4, 38.3)^d^	<0.01
Waist circumference (cm)	91.5 (67.0, 125.0)	91.1 (64.0, 133.0)	90.2 (63.0, 135.0)^d^	89.8 (63.0, 124.0)^d^	<0.001
TC (mmol/L)	5.24 (2.72, 10.06)	5.26 (2.97, 9.96)	5.26 (2.05, 10.42)	5.25 (2.99, 15.45)	0.55
TG (mmol/L)	1.92 (0.41, 19.81)	1.98 (0.32, 16.04)	2.06 (0.37, 17.71)	2.20 (0.36, 28.35)^d^	0.02
LDL (mmol/L)	3.15 (1.09, 7.06)	3.17 (0.81, 7.98)	3.17 (0.63, 8.00)	3.16 (1.19, 10.45)	0.51
HDL (mmol/L)	1.30 (0.30, 2.74)	1.27 (0.62, 2.75)	1.28 (0.51, 2.71)	1.26 (0.46, 2.60)^d^	0.08
SBP (mmHg)	126.6 (90.0, 205.0)	125.9 (90.0, 205.0)	125.3 (85.0, 190.0)	124.2 (80.0, 180.0)^d^	0.03
DBP (mmHg)	82.6 (50.0, 125.0)	82.3 (50.0, 130.0)	81.7 (50.0, 125.0)	81.2 (50.0, 125.0)^d^	0.0504
FBS (mmol/L)	5.45 (3.60, 19.20)	5.32 (3.60, 15.40)	5.36 (3.50, 20.20)	5.28 (3.50, 19.10)^d^	0.07
IgA (mg/dL)	226.5 (8.1, 657.0)	235.3 (51.8, 690.0)	236.1 (41.2, 745.0)	246.3 (6.7, 718.0)^d^	<0.01
IgG (mg/dL)	1090.5 (517.0, 3330.0)	1132.0 (420.0, 2340.0)^d^	1145.0 (447.0, 2020.0)^d^	1187.6 (55.4, 2330.0)^d^	<0.0001
Smoking status (%)					
Smoker	50.8	49.1	46.3	49.4	0.31
Ex-smoker	0.14	0.21	0.36	0.21	0.60
Drinker (%)	68.5	67.2	66.0	65.8	0.11
Family history of diseases (%)					
CVD	33.8	30.3	29.3	27.8	<0.001
Hypertension	46.6	46.4	44.9	43.8	0.09
Hyperlipidemia	0.21	0.64	0.28	0.14	0.30
Diabetes	20.1	19.2	18.2	17.3	0.045

a BMI, body mass index; TC, total cholesterol; TG, triglycerides; LDL, low density lipoprotein cholesterol; HDL, high-density lipoprotein-cholesterol; SBP, systolic blood pressure; DBP, diastolic blood pressure; FBS, fasting blood sugar; UA, uric acid; CVD, cardiovascular disease; Ig, immunoglobulin.

b Analysis of variance or logistic regression analysis.

c Mean (range) (all such values).

d Significantly different from the lowest quartile of immunoglobulin M (Bonferroni correction): *P*<0.05.

**Table 2** presents characteristics of female participants according to quartiles of IgM. Mean age, BMI, waist circumferences, TC, TG, LDL, SBP, DBP, and FBS were significantly lower across IgM quartiles (*P* for trend <0.001). Mean IgG was significantly higher across IgM quartiles (*P* for trend <0.0001). The percentage of subjects with a family history of CVD, and who were current smokers were significantly lower in the highest IgM quartile (*P* for trend <0.01 and 0.001, respectively). No significant differences in HDL, drinking status, family history of hypertension, hyperlipidemia, or diabetes were observed across quartiles of IgM.

**Table 2:** Female participant characteristics by quartiles of immunoglobulin M (n = 3,706)^a^.

	Quartiles of immunoglobulin M (range, mg/dL)				
	Level 1 (7.2–80.8)	Level 2 (80.9–111.0)	Level 3 (112.0–152.0)	Level 4 (153.0–2480.0)	*P* for trend b
	(n = 927)	(n = 942)	(n = 900)	(n = 937)	
Age (y)	51.3 (26.0, 84.0)^c^	47.7 (27.0, 81.0)^d^	45.1 (25.0, 83.0)^d^	44.2 (28.0, 76.0)^d^	<0.0001
BMI (kg/m2)	25.0 (16.4, 45.5)	24.3 (16.0, 36.5)^d^	23.8 (11.6, 40.0)^d^	23.5 (16.2, 36.1)^d^	<0.0001
Waist circumference (cm)	82.3 (60.0, 127.0)	80.4 (56.0, 115.0)^d^	78.4 (51.0, 109.0)^d^	77.6 (57.0, 112.0)^d^	<0.0001
TC (mmol/L)	5.41 (3.10, 8.71)	5.32 (3.09, 9.67)	5.20 (2.80, 10.34)^d^	5.14 (2.89, 11.32)^d^	<0.0001
TG (mmol/L)	1.45 (0.33, 7.05)	1.36 (0.35, 10.27)	1.27 (0.37, 11.07)^d^	1.31 (0.32, 23.74)^d^	<0.0001
LDL (mmol/L)	3.26 (0.70, 6.11)	3.19 (1.35, 7.44)	3.11 (1.12, 7.87)^d^	3.05 (1.17, 7.77)^d^	<0.001
HDL (mmol/L)	1.51 (0.72, 3.17)	1.54 (0.62, 2.94)	1.54 (0.74, 2.86)	1.53 (0.73, 2.99)	0.10
SBP (mmHg)	126.1 (80.0, 200.0)	121.8 (80.0, 185.0)^d^	119.2 (80.0, 190.0)^d^	118.3 (80.0, 180.0)^d^	<0.0001
DBP (mmHg)	77.2 (50.0, 115.0)	75.3 (50.0, 110.0)^d^	74.6 (50.0, 110.0)^d^	74.4 (50.0, 125.0)^d^	<0.0001
FBS (mmol/L)	5.22 (3.30, 22.70)	4.99 (3.60, 14.20)^d^	4.98 (3.40, 14.30)^d^	4.96 (3.30, 16.50)^d^	<0.0001
IgA (mg/dL)	234.5 (51.6, 1230.0)	231.4 (6.7, 651.0)	230.1 (65.3, 716.0)	239.0 (71.4, 1140.0)	0.33
IgG (mg/dL)	1213.6 (637.0, 2490.0)	1229.4 (599.0, 2240.0)	1260.0 (665.0, 2590.0)^d^	1273.7 (49.8, 2650.0)^d^	<0.0001
Smoking status (%)					
Smoker	5.5	6.2	3.8	3.6	<0.01
Ex-smoker	0.00	0.00	0.00	0.00	-
Drinker (%)	13.4	13.8	14.3	14.7	0.37
Family history of diseases (%)					
CVD	34.8	31.5	30.6	27.6	0.001
Hypertension	44.4	46.9	47.3	46.2	0.52
Hyperlipidemia	0.32	0.85	0.22	0.43	0.69
Diabetes	19.2	17.8	18.2	16.4	0.16

a BMI, body mass index; TC, total cholesterol; TG, triglycerides; LDL, low density lipoprotein cholesterol; HDL, high-density lipoprotein-cholesterol; SBP, systolic blood pressure; DBP, diastolic blood pressure; FBS, fasting blood sugar; UA, uric acid; CVD, cardiovascular disease; Ig, immunoglobulin.

b Analysis of variance or logistic regression analysis.

c Mean (range) (all such values).

d Significantly different from the lowest quartile of immunoglobulin M (Bonferroni correction): *P*<0.05.

**Figure 1:**
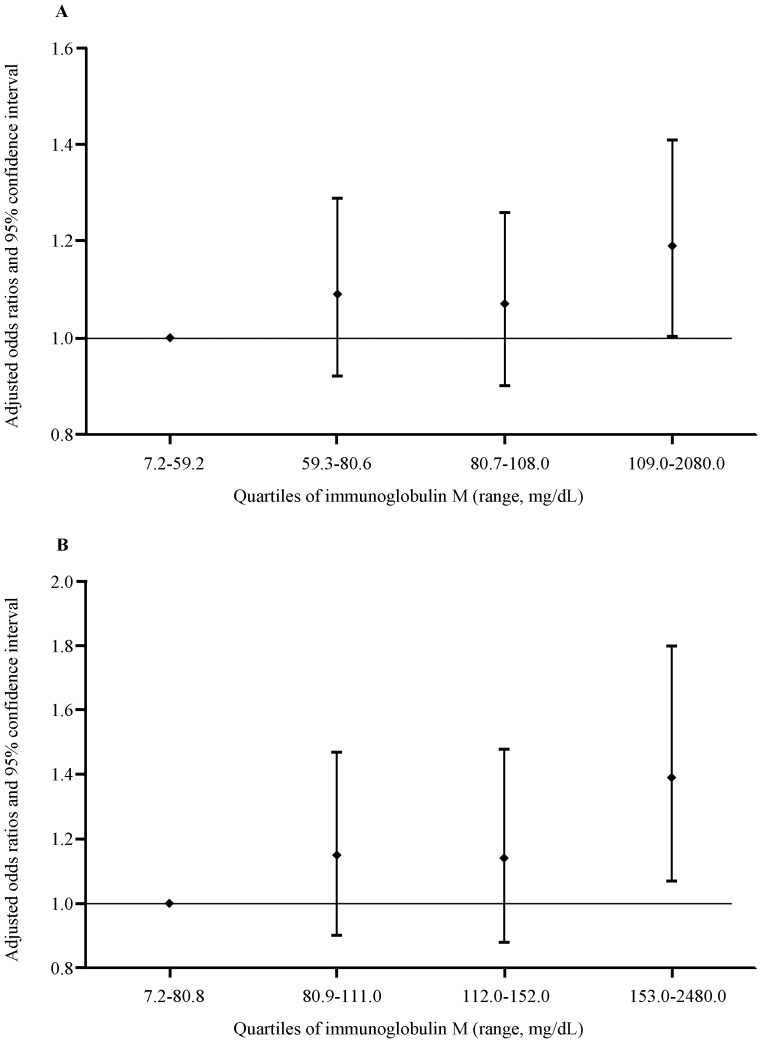
Adjusted odds ratio (95% confidence interval) of the relationship between the quartiles of immunoglobulin M and metabolic syndrome (A, males; B, females). Adjusted for age, body mass index, smoking status, drinking status, and family history of cardiovascular disease, hypertension, hyperlipidemia, and diabetes, and immunoglobulin A, and G.

**Table 3** shows the crude and adjusted relationships between quartiles of IgM and MS and its components in male participants. In the final multivariate models, the adjusted OR (95% CI) for MS across IgM quartiles were 1.00 (reference), 1.09 (0.92, 1.29), 1.07 (0.90, 1.26), and 1.19 (1.002, 1.41) (*P* for trend  = 0.07), respectively. Similar results also were observed in females (**Table 4**). The **figure 1** visualizes the relationship between the quartiles of IgM and MS (A, males; B, females). Furthermore, multivariable models were developed using stepwise variable selection. There are almost no changes for the adjusted ORs and 95% CI both in males and females.

**Table 3:** Adjusted relationships of quartiles of immunoglobulin M to metabolic syndrome (males, n = 5,673)^a^.

	Quartiles of immunoglobulin M (range, mg/dL)				
	Level 1 (7.2–59.2)	Level 2 (59.3–80.6)	Level 3 (80.7–108.0)	Level 4 (109.0–2080.0)	*P* for trend^b^
	(n = 1,422)	(n = 1,415)	(n = 1,406)	(n = 1,430)	
No. of Metabolic syndrome (presence of any 3 of 5 risk factors)	633	618	584	605	-
Crude	Reference	0.97 (0.83, 1.12)^c^	0.89 (0.76, 1.03)	0.91 (0.79, 1.06)	0.16
Age-adjusted	Reference	1.03 (0.89, 1.19)	0.94 (0.81, 1.10)	0.98 (0.84, 1.14)	0.54
Age- and BMI-adjusted	Reference	1.05 (0.89, 1.24)	1.02 (0.87, 1.21)	1.12 (0.95, 1.33)	0.21
Multiple adjusted^d^	Reference	1.08 (0.91, 1.27)	1.05 (0.89, 1.24)	1.15 (0.98, 1.36)	0.12
Multiple adjusted^e^	Reference	1.09 (0.92, 1.29)	1.07 (0.90, 1.26)	1.19 (1.002, 1.41)	0.07
					
Metabolic syndrome components					
No. of elevated waist circumference (≥85 cm in males; ≥80 cm in females)	1,130	1,104	1,030	1,037	-
Crude	Reference	0.92 (0.77, 1.10)	0.71 (0.59, 0.84)	0.68 (0.57, 0.81)	<0.0001
Age-adjusted	Reference	1.00 (0.83, 1.20)	0.77 (0.64, 0.92)	0.75 (0.63, 0.89)	<0.0001
Age- and BMI-adjusted	Reference	1.04 (0.80, 1.35)	0.77 (0.59, 0.99)	0.86 (0.67, 1.11)	0.08
Multiple adjusted^d^	Reference	1.07 (0.82, 1.39)	0.77 (0.60, 1.00)	0.87 (0.67, 1.13)	0.08
Multiple adjusted^e^	Reference	1.09 (0.84, 1.42)	0.79 (0.61, 1.02)	0.90 (0.69, 1.17)	0.13
No. of elevated triglycerides (≥1.7 mmol/L)	640	676	665	703	-
Crude	Reference	1.12 (0.96, 1.30)	1.10 (0.95, 1.27)	1.18 (1.02, 1.37)	0.047
Age-adjusted	Reference	1.10 (0.95, 1.28)	1.08 (0.93, 1.26)	1.17 (1.01, 1.35)	0.07
Age- and BMI-adjusted	Reference	1.12 (0.96, 1.31)	1.15 (0.99, 1.35)	1.29 (1.10, 1.50)	<0.01
Multiple adjusted^d^	Reference	1.14 (0.98, 1.33)	1.19 (1.02, 1.39)	1.32 (1.13, 1.54)	<0.001
Multiple adjusted^e^	Reference	1.16 (0.99, 1.36)	1.22 (1.04, 1.42)	1.38 (1.17, 1.61)	0.0001
No. of reduced HDL (<1.0 mmol/L in males; <1.3 mmol/L in females)	238	265	245	281	-
Crude	Reference	1.15 (0.95, 1.39)	1.05 (0.86, 1.28)	1.22 (1.01, 1.47)	0.10
Age-adjusted	Reference	1.12 (0.92, 1.36)	1.03 (0.84, 1.25)	1.19 (0.98, 1.44)	0.16
Age- and BMI-adjusted	Reference	1.13 (0.93, 1.38)	1.08 (0.88, 1.32)	1.27 (1.05, 1.55)	0.03
Multiple adjusted^d^	Reference	1.15 (0.94, 1.40)	1.10 (0.90, 1.35)	1.29 (1.06, 1.57)	0.02
Multiple adjusted^e^	Reference	1.12 (0.92, 1.37)	1.07 (0.88, 1.31)	1.22 (1.00, 1.49)	0.09
No. of elevated blood pressure (systolic: ≥130 and/or diastolic: ≥85 mmHg)	806	756	718	704	-
Crude	Reference	0.88 (0.76, 1.02)	0.80 (0.69, 0.93)	0.74 (0.64, 0.86)	<0.0001
Age-adjusted	Reference	0.98 (0.84, 1.14)	0.89 (0.77, 1.04)	0.84 (0.72, 0.97)	<0.01
Age- and BMI-adjusted	Reference	0.99 (0.84, 1.16)	0.94 (0.80, 1.10)	0.90 (0.77, 1.06)	0.15
Multiple adjusted^d^	Reference	0.99 (0.85, 1.17)	0.94 (0.80, 1.10)	0.92 (0.78, 1.07)	0.21
Multiple adjusted^e^	Reference	0.98 (0.84, 1.16)	0.93 (0.79, 1.09)	0.90 (0.76, 1.05)	0.13
No. of elevated fasting glucose (≥5.56 mmol/L)	416	371	369	365	-
Crude	Reference	0.86 (0.73, 1.01)	0.86 (0.73, 1.02)	0.83 (0.70, 0.98)	0.04
Age-adjusted	Reference	0.95 (0.80, 1.12)	0.95 (0.81, 1.13)	0.92 (0.78, 1.09)	0.39
Age- and BMI-adjusted	Reference	0.96 (0.81, 1.14)	1.00 (0.84, 1.18)	0.99 (0.83, 1.17)	0.98
Multiple adjusted^d^	Reference	0.98 (0.83, 1.17)	1.03 (0.86, 1.22)	1.02 (0.86, 1.21)	0.72
Multiple adjusted^e^	Reference	1.01 (0.85, 1.2)	1.06 (0.89, 1.26)	1.07 (0.9, 1.28)	0.36

a BMI, body mass index; HDL, high-density lipoprotein-cholesterol.

b Multiple logistic regression analysis.

c Adjusted odds ratios (95% confidence interval) (all such values).

d Adjusted for age, BMI, smoking status, drinking status, and family history of cardiovascular disease, hypertension, hyperlipidemia, and diabetes.

e Adjusted for age, BMI, smoking status, drinking status, and family history of cardiovascular disease, hypertension, hyperlipidemia, and diabetes, and immunoglobulin A, and G.

**Table 4:** Adjusted relationships of quartiles of immunoglobulin M to metabolic syndrome (females, n = 3,706)^a^.

	Quartiles of immunoglobulin M (range, mg/dL)				
	Level 1 (7.2–80.8)	Level 2 (80.9–111.0)	Level 3 (112.0–152.0)	Level 4 (153.0–2480.0)	*P* for trend^b^
	(n = 927)	(n = 942)	(n = 900)	(n = 937)	
No. of Metabolic syndrome (presence of any 3 of 5 risk factors)	258	225	171	183	-
Crude	Reference	0.81 (0.66, 1.00)^c^	0.61 (0.49, 0.76)	0.63 (0.51, 0.78)	<0.0001
Age-adjusted	Reference	1.05 (0.84, 1.32)	0.93 (0.73, 1.18)	1.05 (0.83, 1.33)	0.90
Age- and BMI-adjusted	Reference	1.16 (0.91, 1.47)	1.14 (0.88, 1.48)	1.40 (1.08, 1.81)	0.02
Multiple adjusted^d^	Reference	1.15 (0.90, 1.47)	1.14 (0.88, 1.48)	1.40 (1.08, 1.81)	0.02
Multiple adjusted^e^	Reference	1.15 (0.90, 1.47)	1.14 (0.88, 1.48)	1.39 (1.07, 1.80)	0.02
Metabolic syndrome components					
No. of elevated waist circumference (≥85 cm in males; ≥80 cm in females)	538	490	368	376	-
Crude	Reference	0.78 (0.65, 0.94)	0.50 (0.42, 0.60)	0.49 (0.40, 0.58)	<0.0001
Age-adjusted	Reference	0.99 (0.81, 1.21)	0.72 (0.59, 0.88)	0.75 (0.61, 0.91)	<0.001
Age- and BMI-adjusted	Reference	1.22 (0.95, 1.57)	0.88 (0.68, 1.14)	1.07 (0.82, 1.39)	0.74
Multiple adjusted^d^	Reference	1.21 (0.94, 1.57)	0.88 (0.68, 1.14)	1.06 (0.82, 1.38)	0.71
Multiple adjusted^e^	Reference	1.22 (0.94, 1.57)	0.88 (0.68, 1.14)	1.07 (0.82, 1.39)	0.74
No. of elevated triglycerides (≥1.7 mmol/L)	255	209	166	191	-
Crude	Reference	0.75 (0.61, 0.93)	0.60 (0.48, 0.74)	0.68 (0.54, 0.84)	<0.001
Age-adjusted	Reference	0.89 (0.71, 1.11)	0.79 (0.63, 1.00)	0.95 (0.76, 1.19)	0.59
Age- and BMI-adjusted	Reference	0.93 (0.74, 1.16)	0.88 (0.70, 1.12)	1.11 (0.88, 1.40)	0.43
Multiple adjusted^d^	Reference	0.94 (0.75, 1.17)	0.90 (0.71, 1.14)	1.14 (0.90, 1.44)	0.30
Multiple adjusted^e^	Reference	0.94 (0.75, 1.18)	0.90 (0.71, 1.15)	1.15 (0.91, 1.46)	0.27
No. of reduced HDL (<1.0 mmol/L in males; <1.3 mmol/L in females)	262	250	229	262	-
Crude	Reference	0.92 (0.75, 1.12)	0.87 (0.70, 1.07)	0.99 (0.81, 1.21)	0.89
Age-adjusted	Reference	0.93 (0.76, 1.14)	0.89 (0.72, 1.10)	1.02 (0.83, 1.25)	0.84
Age- and BMI-adjusted	Reference	0.98 (0.79, 1.21)	0.99 (0.79, 1.23)	1.16 (0.94, 1.44)	0.14
Multiple adjusted^d^	Reference	0.98 (0.79, 1.21)	0.99 (0.80, 1.23)	1.17 (0.94, 1.45)	0.13
Multiple adjusted^e^	Reference	0.97 (0.78, 1.20)	0.96 (0.77, 1.20)	1.11 (0.90, 1.38)	0.30
No. of elevated blood pressure (systolic: ≥130 and/or diastolic: ≥85 mmHg)	431	357	289	275	-
Crude	Reference	0.70 (0.58, 0.84)	0.54 (0.45, 0.66)	0.48 (0.40, 0.58)	<0.0001
Age-adjusted	Reference	0.95 (0.77, 1.18)	0.93 (0.75, 1.17)	0.89 (0.71, 1.11)	0.29
Age- and BMI-adjusted	Reference	1.01 (0.81, 1.26)	1.05 (0.83, 1.31)	1.04 (0.83, 1.31)	0.69
Multiple adjusted^d^	Reference	1.01 (0.81, 1.26)	1.03 (0.82, 1.30)	1.03 (0.82, 1.30)	0.78
Multiple adjusted^e^	Reference	1.00 (0.80, 1.25)	1.02 (0.81, 1.28)	1.00 (0.79, 1.26)	0.97
No. of elevated fasting glucose (≥5.56 mmol/L)	195	141	123	122	-
Crude	Reference	0.66 (0.52, 0.84)	0.59 (0.46, 0.76)	0.56 (0.44, 0.72)	<0.0001
Age-adjusted	Reference	0.81 (0.63, 1.04)	0.86 (0.66, 1.11)	0.87 (0.67, 1.13)	0.40
Age- and BMI-adjusted	Reference	0.84 (0.65, 1.08)	0.94 (0.72, 1.22)	0.98 (0.75, 1.27)	0.92
Multiple adjusted^d^	Reference	0.85 (0.66, 1.09)	0.95 (0.73, 1.24)	1.00 (0.77, 1.31)	0.77
Multiple adjusted^e^	Reference	0.85 (0.65, 1.09)	0.95 (0.73, 1.24)	0.99 (0.75, 1.29)	0.87

a BMI, body mass index; HDL, high-density lipoprotein-cholesterol.

b Multiple logistic regression analysis .

c Adjusted odds ratios (95% confidence interval) (all such values).

d Adjusted for age, BMI, smoking status, drinking status, and family history of cardiovascular disease, hypertension, hyperlipidemia, and diabetes.

e Adjusted for age, BMI, smoking status, drinking status, and family history of cardiovascular disease, hypertension, hyperlipidemia, and diabetes, and immunoglobulin A, and G.

In the MS components analysis, elevated TG was positively related to IgM quartiles (*P* for trend  = 0.0001) in male, in the final model (**Table 3**). Although the difference was not statistically significant (*P* for trend  = 0.09), the highest proportion of participants with reduced HDL was in the highest IgM quartile (OR [95% CI]: 1.22 [1.00, 1.49]) (**Table 3**). In contrast, no significant relations were found between IgM quartiles and other MS components in the final multivariate models in females (**Table 4**).

## Discussion

In this cross-sectional study, we have investigated the relationships between levels of IgM concentration and MS in an adult population. This study is the first to show that the highest immunoglobulin M quartile is independently related to the highest prevalence of MS in both males and females. Furthermore, in males IgM levels were positively and independently related to the prevalence of elevated TG and reduced HDL.

No previous studies have indicated that serum IgM levels were positively and significantly related to MS among the general population. A small-scale cross-sectional study has investigated the relationships between serum immunoglobulin concentrations and MS among a population of 460 adults [21]. In contrast to our results, this study found no significant relationship between IgM and MS after adjustment for age, sex, smoking, and drinking status. Although the reason remains unclear, differences in adjustment factors and population size may partly explain the discrepancy. As in our male population, this study also found that IgM levels were related to elevated TG and reduced HDL. We therefore speculate that lipid metabolism disorder may be a key point in the link between serum IgM levels and MS among the general population. More interestingly, it was found that IgM levels were not related to elevated waist circumference, and were negatively related to BMI both in previous studies [21], [24] and in our observations, suggesting that alteration in TG and HDL metabolism is more important in the relationships between IgM and MS than pure abdominal adiposity or increased BMI.

Several experimental studies have consistently pointed to serum fatty acid as an important inducing molecule of the innate immune system through activation of toll-like receptors (TLR) [25]. Recent studies have also suggested that fatty acid activates B cell TLR4, and that there is a requirement for B cell TLR stimulation by fatty acid for increased IgM [14], [26]. Because TG provides the source of fatty acid needed throughout body cells, and both are closely related, serum fatty acid concentration may help in the interpretation of our observations of the relationships between IgM and MS, TG, or HDL. However, due to our lack of data on serum fatty acid composition, we cannot adequately determine whether fatty acid is a more accurate molecule for explaining our observations. Further research is warranted to clarify the causality and precise mechanisms between IgM and lipid metabolism disorder, especially hypertriglyceridemia and/or elevated serum fatty acid composition.

In contrast to our observations, a previous study has shown that elevated levels of IgA, IgE, and IgG, but not IgM, are related to myocardial infarction and cardiac death in males with dyslipidemia [27]. It is noteworthy, however, that the baseline TG levels were similar between cases and controls in this case-control study (see textual Table 1). Because our results showed that IgM was strongly related to elevated TG, the difference in serum TG concentrations among groups might be a cause for the observational discrepancy.

Consistent with previous studies, our study also found that females have higher IgM levels than males [21]. Although the precise mechanism remains unclear, the stimulatory action of estrogens on B lymphocytes could be the cause [28]. It is interesting to note that the prevalence of MS is lower in females (it is also observed in other populations [29]), while IgM levels are higher, suggesting a gender-specific relationship between IgM and MS. Although the analysis of individual components of MS showed that serum IgM levels were mainly related to elevated TG in both males and females, the relationship is not statistically significant in females (females had the largest OR in the highest quartile as compared to other individual components of MS). Because TG levels are significantly higher in males (geometric mean, [95% CI]: 1.69 [1.66–1.71] compared with 1.16 [1.14–1.18] mmol/L, *P*<0.0001), differences in TG levels may partly explain these observations. Furthermore, despite being positively and significantly related to MS in females, IgM levels were not significantly related to any one of the individual components of MS. We speculate that in females, IgM may be strongly related to a constellation of risk factors rather than a simple risk factor.

A large number of studies have established that most autoimmune diseases occur significantly more frequently in females than males [30]. Several mechanisms such as X chromosome inactivation, the effects of sex hormones on immune function, fetal–maternal microchimerism, and redox state etc. have been proposed as explanations for this gender bias [30]–[32]. The present results showed that elevated levels of TG and low levels of HDL correlated with reduced levels of IgM in males, and that TG levels are significantly higher in males than females, implying the effect of TG on IgM may depend on TG levels, but not gender. Further studies are required to identify the hypothesis.

It is increasingly recognized that MS is a low-grade inflammatory condition and central abdominal fat is a contributing factor [33]. The molecular mechanisms that contribute to this inflammation are still quite unclear. Inflammation of central adipose tissue leads to adipokine production, followed by secretion of adipokines into the general circulation contributing to the overall inflammatory condition [34]. An increased accumulation of macrophages occurring in obese adipose tissue has emerged as a key process in metabolic inflammation [6]. The heterogeneity of adipose tissue macrophages and their physical and functional interactions with adipocytes, endothelial cells, and other immune cells within the adipose tissue microenvironment is recognized in playing a critical role in the development and progression of inflammation [6]. Furthermore, up-regulation of TLR4/nuclear factor-kappaB is considered to be major signaling pathways [34], [35]. However, few studies have investigated the question of whether IgM has an influence on the pathological process of MS. The present study provides a novel viewpoint that IgM may be involved in the pathological process of MS.

The present study has two limitations. Firstly, because this is a cross-sectional study, further prospective studies and intervention trials should be undertaken to establish a causal relationship between IgM and MS. Secondly, although we adjusted for a considerable number of potentially confounding factors, we cannot exclude the possibility that MS is affected by other lifestyle variables which are intrinsically related to serum IgM concentration.

This large-scale epidemiological study has shown that the highest immunoglobulin M quartile is independently related to the highest prevalence of MS in males. But, IgM only was significantly related to elevated TG and reduced HDL, but not waist circumference, BP and FBS. This suggests that IgM may be involved in the pathological process of MS through lipid metabolism disorder. In females, serum IgM levels were independently related to a higher prevalence of MS, but not its components, suggesting IgM may be strongly related to a constellation of risk factors rather than a simple risk factor. To conclude, this study is the first to show that IgM is independently related to MS and its individual components (elevated triglycerides and reduced high-density lipoprotein cholesterol) in males, whereas IgM is independently related to MS in females but not to its individual components. The present study also implied that IgM might be a useful predictive factor for MS in an adult population. Further studies are needed to explore the causality and exact mechanisms of IgM in MS.
